# Reply to Meshkini et al.

**DOI:** 10.1038/s41430-021-00929-y

**Published:** 2021-05-13

**Authors:** Armin Zittermann, Heiner K. Berthold, Stefan Pilz

**Affiliations:** 1 Clinic for Thoracic and Cardiovascular Surgery, Herz- und Diabeteszentrum NRW, Ruhr University Bochum, Bad Oeynhausen, Germany; 2 Department of Internal Medicine and Geriatrics, Bethel Clinic (EvKB), Bielefeld, Germany; 3 Division of Endocrinology and Diabetology, Department of Internal Medicine, Medical University of Graz, Graz, Austria

**Keywords:** Endocrine system and metabolic diseases, Biomarkers

## To the Editor:

Meshkini et al. [1] raised several issues regarding our meta-analysis [2]. With respect to the exclusion of specific age groups, we disagree that children should be deleted from data analysis. Fibroblast growth factor 23 (FGF-23) is a cardiovascular risk marker. Cardiovascular disease is a chronic illness and cardiovascular lesions can already occur during childhood and adolescence. Moreover, there are no major differences in vitamin D metabolism between adults and children at the average age included in our meta-analysis. Since we included only RCTs with a placebo group, each age group had its own control. Anyway, a sensitivity analysis with exclusion of studies including children did not alter our main findings (vitamin D effect on FGF-23: +25 pg/ml [95% CI: 15–34 pg/mL; *P* < 0.001]). Likewise, we are well aware that in case of several treatment groups the number of individuals in the control group has to be divided by the number of treatment groups, when analyzed in meta-analysis, and this is what we did. A subgroup analysis of studies with mean baseline circulating 25-hydroxyvitamin D concentrations below and above 50 nmol/l may indeed lead to misclassification of some individuals. Nevertheless, this kind of classification results in two clearly distinct subgroups and is thus an established methodological approach in case that individual participant data are not available. Notably, our results are in line with another meta-analysis, which reported a significant increase in FGF-23 concentrations by vitamin D supplementation in individuals with initial 25-hydroxyvitamin D concentrations below 50 nmol/l [3].

Regarding missing of eligible RCTs for our meta-analysis, it is challenging to identify all relevant articles, given the large number of vitamin D publications each year and in particular if FGF-23 is not mentioned in the Abstract so that such articles are missed even by a properly performed systematic literature search. Of the six additional studies mentioned by Meshkini et al., two [4, 5] did not provide all baseline or in-study data that were needed for our meta-analysis. Inclusion of the other four studies [6–9] does not substantially change our overall results (vitamin D effect on FGF-23: +19 pg/ml [95% CI: 14–25 pg/mL; *P* < 0.001]) and thus further strengthens the reliability of our meta-analysis. However, we very much appreciate that Meshkini et al. pointed to a misclassification by us of daily vitamin D dosing in the included study by Trummer et al. (2800 IU daily instead of 2000 IU daily) [10]. We apologize for this mistake and re-analyzed and re-classified our data (subgroups: daily vitamin D dose equivalent up to 3000 IU; daily vitamin D dose equivalent >3000 IU daily; administration of activated vitamin D) and also included the four aforementioned studies by Meshkini et al. [6–9]. The results remain similar to the ones of our article [2] (vitamin D dose equivalent ≤3000 IU/day: +1 pg/ml [95% CI: 1–2 pg/ml]; vitamin D dose equivalent >3000 IU/day: +16 pg/ml [95% CI: 4–28 pg/ml]; administration of activated vitamin D: +67 pg/ml [95% CI: 16–117 pg/ml]; *P*_interaction_ < 0.001), except that our re-analysis now shows that daily vitamin D doses up to 3000 IU instead of up to 2000 IU vitamin D daily do not result in a substantial change in FGF-23.

Altogether, the vitamin D dose re-classification in one study results in the new conclusion that vitamin doses up to 3000 IU daily do not influence FGF-23 concentrations. However, all of our other conclusions do not change substantially so that the reliability of our main findings is further supported by the additional analyses due to the comments raised by Meshkini et al.
